# Mr. Christie on Dysentery

**Published:** 1799-06

**Authors:** J. Christie

**Affiliations:** 27th Foot. 26, Northumberland-street


GENTLEMEN,

AS there is among practitioners considerable difference in opinion, with respect to the nature and causes of dysentery, and great variety in its appearance, as well as treatment in different climates; and as especially the method so successfully employed throughout the East-Indies, of giving mercury in the cure of this disease, may not perhaps be generally known, but which, however, in obstinate cases may be more frequently used with advantage in other countries also,—I have ventured to transmit you some observations I have have had occaſion to make in this diſeaſe; in which I have alſo committed ſome thoughts on the nature of contagion in general; and, ſhould theſe be found not altogether unworthy of your conſiderations, I hope you will do me the honor of inſerting them in your uſeful Journal.—As the cure of dyſentery ſeems now in general to be truſted almoſt entirely to the uſe of proper laxatives, I ſhall here forbear making any obſervations on the treatment of dyſentery, which I have had frequent opportunities of ſeeing in this country, but confine myſelf chiefly to the diſeaſe as it appeared in a detachment of an European regiment, on their arrival in the Eaſt-Indies, in 1796. To this I ſhall add ſome account of the appearances on the diſſection of thoſe who died of the complaint.

I am, Gentlemen, with the greateſt reſpect,

26, *Northumberland-ſtreet,* Your moſt obedient ſervant,

30*th April,* 1799. J. Christie. 27th Foot.

In May, 1796, a detachment, of which I had the charge, conſiſting of 320 men, of his majeſty's nineteenth regiment of foot, were embarked at Portſmouth for the Eaſt-Indies; the greater part of thoſe ſoldiers were young men under the age of twenty-one, all were healthy, and few had ever been in a hot climate.

After a remarkable fine paſſage from England, we arrived at Bombay in September following, two men only having been buried during the paſſage, one of whom died of Ileus, the other of tetanus, ariſing from a compound fracture, and for which opium in large doſes, together with calomel, were unſuccefsfully employed; two men were landed ill of flux, and ſeveral had ſymptoms of ſcurvy, of which they got rapidly clear by proper diet, on their arrival at Bombay.

This detachment on landing, along with a company conſiſting of an hundred men of the thirty-third regiment, were put into clean, ſpacious, and airy barracks, ſituated on a ſmall iſland, or rather peninſula, almoſt entirely ſurrounded by the ſea, and diſtant a mile from Bombay, to which, at low water, there is a communication for foot paſſengers.

The men were ſupplied with bedding and light cloathing fit for the climate, and more than uſual care and attention was paid by the commanding officer to the diet, regularity, and diſcipline; whatever could tend to preſerve the health and comfort of this detachment, now conſiſting of upwards of 400, was enforced by the ſtricteſt orders: notwithſtanding all which, ſoon after our arrival, in the beginning of September, at the breaking ing up of the monſoon on the coaſt of Malabar, ſymptoms of dyſentery, chiefly among the youngeſt and ſtouteſt, became very general.

The diſeaſe attacked the perſon with the uſual ſymptoms of frequent ſtools and ſevere griping: theſe ſtools, ſmall in quantity, having a ſcybalous appearance, and conſtantly mixed with blood. Coſtiveneſs, though very rarely, preceded the diſeaſe. The griping, teneſmus, and purging, were always accompanied with fever, apparently inflammatory. The ſkin was hot and parched, thirſt great, pulſe frequent, ſtrong and hard, generally beating from 100 to 120 ſtrokes in the minute, but with that peculiar ſmallneſs or wiry feel which characteriſes viſceral from other inflammatory affections; and although this diſeaſe is in general ſuppoſed to be kept up by ſpaſmodic derangement, I would ſay, from what I have obſerved in practice, and particularly from the appearances on diſſection, that this diſeaſe is generally, and when violent, conſtantly, uſhered in with more or leſs inflammatory action. That type was perhaps the more remarkably obſerved at this period, from the diſeaſe having attacked chiefly the young and plethoric, and thoſe of the moſt robuſt conſtitutions.

Soon after the appearance of the diſeaſe, generally about the third or fourth day, and always, when violent, the patient complained of a ſcalding, and difficulty in voiding urine. In ſome, this continued throughout the diſeaſe, with little inconvenience; in others, thoſe ſymptoms increaſed ſo much, that a complete ſuppreſſion of urine, requiring the uſe of the catheter, took place. In general, the violence of the ſtrangury was the ſureſt ſign of that of the diſeaſe, and few recovered wherein this ſymptom was obſtinate *.

The teneſmus was uncommonly diſtreſſing, and ſo little was voided, that the patient had generally upwards of thirty ſtools in the courſe of a day, and in ſome, two or three in one hour.

Although the fever at firſt was evidently of inflammatory tendency, yet it was ſoon followed by great proſtration of ſtrength, accompanied with early marks of general putreſcency.

In

---

* Some have supposed dysuria dysenterica to arise in consequence of a portion of the rectum protruding by the severe tenesmus, and thus carrying along with it, in some measure, the fundus of the bladder, to which it is connected by cellular substance. In this manner they suppose the mouth of the bladder is mechanically elevated and moved from the direct entrance of the urethra, occasioning strangury.— It appears probable to me, however, that some degree of inflammation is communicated from the rectum to the neck of the bladder, and the urine itself appears also to be more than usually acrimonious, and will thereby give rise to the symptom mentioned.

In moſt of the more violent caſes, there was great oppreſſion at the præcordia, attended by nauſea and vomiting; in one or two inſtances there was a voracious appetite.

The diſeaſe ſeldom aſſumed the appearance of what writers have called *mucoſus*, or *dyſenteria alba:* when it appeared in this form, it was generally a mark of returning health.

At times, the matter voided appeared to be pure blood, but generally the ſtools appeared to be mixed with a purulent-like matter, or a kind of ſanies, and this, from its acrimony, produced great diſtreſs, eſpecially the giving riſe to very troubleſome ſores around the anus: the ſtools had always an unuſually fœtid odour, and indeed the ſmell of the whole body was remarkably diſagreeable. Worms of the lumbrical kind were ſometimes voided; and, in one inſtance, a large one crawled out by the mouth. In the ſtools there was frequently obſerved a ſuety or fatty-like appearance, but moſt commonly films and portions of a membranous ſubſtance were voided; and, when the diſeaſe was of long continuance eſpecially, it was aſtoniſhing how large the portions of membrane were that came away in this manner.—I have ſeen pieces at leaſt two or three feet in length, ſometimes cylindrical, reſembling a ſmall inteſtine. It appeared to me, as if the inner membrane of the gut had ſloughed off; but I muſt here acknowledge that I did not examine this appearance with that minuteneſs, to aſcertain whether it was really portions of the villous coat, or merely coagulable lymph, poured out from the abraded ſurface of the inteſtine, and aſſuming the appearance mentioned. I know that ſmall portions of the inner coat of the inteſtines are occaſionally voided in dyſentery in this manner, but as this is doubted by ſome, and denied by others, I have now only to regret not having paid more attention to this matter, for in India particularly, ſuch appearances are by no means unfrequent. It is, however, I think moſt probable, that it is coagulable lymph chiefly, for it ſeemed to retard but little the recovery of the patient.

In many inſtances, the febrile ſtate was moderate, and ſometimes entirely diſappeared, while the proper dyſenteric ſymptoms continued; at other times the fever was kept up, and not unfrequently accompanied with a dull, heavy kind of pain in the right hypochondrium, increaſing on preſſure, and extending towards the clavicle, plainly ſhewing the diſeaſe to be accompanied with a diſordered ſtate of the liver; and in ſuch, a diarrhœa was often obſerved to continue long after the other dyſenteric ſymptoms diſappeared.

By ſome it has been doubted whether the pain referred to the ſhoulder and clavicle, ſo conſtant in hepatitis, and ſo frequent in dyſentery, ſhould be aſcribed to nervous ſympathy.—I ſhould ſuppoſe there can be little doubt of this being a nervous affection; and the peculiar feel may be occasioned by the mere bulk and weight acquired by the liver, in its diseased state; for the pains are sometimes distinctly referred to the side of the neck, though most commonly to the clavicle, or tip of the shoulder; but we know a considerable branch of the *par vagum*, on entering the thorax, pretty constantly turns round the subclavian artery, and which sends some branches to those parts complained of in liver diseases. If the stomach, intestines, or pancreas, which are also supplied with nerves from the *par vagum* and the intercostals, were, during their diseased changes, to acquire a proportionate enlargement and weight, as in the liver, it is probable we should, on mere mechanical principles, perceive similar pains in those viscera.

The dysentery, in most cases, began gradually to become less violent in ten or twelve days from the first attack, and would entirely disappear about the fourteenth or fifteenth. In others, all the symptoms would continue with great severity for weeks together; petechiæ would appear on the skin, and the patient becoming weaker and weaker, would sink under all the appearances of putrid fever. In one or two instances, where the inflammatory symptoms ran unusually high, and accompanied with painful and obstinate strangury, the patients were carried off delirious, on the fourth or fifth day of the disease.

Those who had relapses had the disease most severely, and if the attacks were more than once repeated, few entirely recovered: most of those had some affection of the liver; and here it may be proper to remark, that this organ may be sometimes materially changed, nay, even matter formed in its substance, without ever producing much pain, or shewing any evident external mark.

Causes.—It has been generally alledged and admitted, that dysentery arises from the action of a specific contagion; of the constant presence of which, however, I have long had my doubts, and especially in those cases which were under my care at Bombay. In all, I could trace the disease to have arisen either after intemperance, great fatigue, exposure to, or sleeping in cold wet night air, especially when in a state of intoxication: whether any of these causes, separately or combined, can ever alone produce the disease, or whether they act merely as debilitating causes, thereby favouring the action of the contagion, I cannot take upon me to determine; although for the reasons already mentioned—from its seldom attacking the temperate and well cloathed, and especially from never having seen the disease under common circumstances, distinctly communicated from one person to another —I believe the former is in most instances more probable. At Bombay, the bodies bodies of many were in a very putrefcent ftate, the feculent matter of all had an unufually offenfive fmell, and yet the difeafe never fhewed any figns of a contagious nature.

In Europe, in crowded tranfports, and in fome hofpitals, I have obferved dyfentery affume a contagious appearance: there, however, it feemed to be from accidental circumftances; as the impurity of very confined air—want of cleanlinefs—want of proper reft—from the bad fmell of the patients already labouring under the difeafe—and efpecially from the highly offenfive feculent matter, acting as a ftimulus on the alimentary canal; fuch circumftances, particularly in hot climates, and acting on exhaufted and intemperate conftitutions, moft commonly feem to be the fole caufe of genuine dyfentery. In hot climates, particularly in intemperate men, where we find the circulation always confiderably accelerated, and debility induced, may not an accumulation of blood be thereby produced in the mefenteric veins, unaided by valves, and otherwife little fupported, thus occafion fome diforder in the bowels; and as the great gut is found principally to be the feat of the difeafe, may not this hypothefis be efpecially applicable to it, from the fmall number of its abforbents?—The more highly acid fæces, however, in the colon, appears to be the principal reafon of its fuffering moft materially.

Among accidental caufes, independent of contagion, producing dyfentery, we may mention an inftance ftated by Monf. Lasonne, of the Royal Society of Medicine, at Paris, where in the year 1749 a difeafe raged among cattle, that died in great numbers, many of whom were buried carelefsly near the houfe of L'Infant Jefus, where numbers of the women in it became affected with dyfentery, and other putrid difeafes, from the effluvium acting as a ftimulus on the alimentary canal.

At Bombay, thofe addicted to intoxication with toddy* and arrack, were fo generally obferved to be attacked with dyfentery, that any perfon not at all conver-

* Toddy is the common name in India for a liquor obtained from the cocoa-nut tree. It is not that mild, sweetish, watery fluid, found in the inside of the nut, but a juice, collected by placing a jar, or kind of receiver, at the top of the tree, around the cut extremity of a peduncle, which produces a bunch of cocoa-nuts. The juice which would have been sent to nourish a bunch of these nuts, is thus collected into the receiver, and when taken down in the mornings, is a pleasantly mild sweet liquor, somewhat resembling new ale; and is in this state frequently taken by the East Indians as a pleasant morning draught. It is gently laxative, but after the heat of the day it ferments, and then becomes very intoxicating, and disposed to affect particularly the alimentary canal. In its fermenting state, the soldiers are very fond of it. From this liquor the best arrack is distilled: and these two liquors are the great bane of the European in India.

conversant in medicine, would easily foretel to a certainty, that those who were reeling about in the sun under the influence of this liquor, would be laid up next day with flux. The toddy alone, from its affecting peculiarly the stomach and bowels, seems to me sufficient, in many instances, to produce the disease; but this, as well as all intoxicating liquors, by the general excitement produced in the system, appears to affect and change the biliary secretion, thus acquiring a higher degree of acrimony, and perhaps in this way assists in aggravating or keeping up the disease.

That great fatigue under the exposure of a burning sun—the drinking large draughts of cold or bad water, when the body is much heated—sleeping (particularly in a state of intoxication) in night or damp air, are, indeed, not only capable of producing this disease, and inflammatory affections and fever, but, in some instances, actually to kill, we have many and various well-authenticated accounts; especially in Dr. Currie's 'Medical Reports,' in his quotation from Quintus Curtius, wherein the army of Alexander the Great suffered so much in passing through a very sterile, sandy country, destitute of water, but which (after long thirst and great fatigue) being obtained, the soldiers drank so greedily, that many died in consequence almost instantly.

About the year 1780, at the time military operations were carrying on in India, against Hyder Ally and the French, when some Highland regiments were sent to that quarter of the world, we find several very striking and melancholy examples of great heat and fatigue on European constitutions; the 72d regiment in particular, soon after their arrival in India, in one march, left behind them two hundred men; many of these expired almost instantly, through mere oppression; the greater number of the rest died of fluxes, and fever in consequence.

In June 1794, at the time I accompanied the army under the command of Earl Moira, which disembarked at Ostend, for the purpose of forming a junction with that under the command of his Royal Highness the Duke of York, in Flanders, we had excessively long and fatiguing marches between Ostend and Ghent. The weather was unusually sultry;—several of the soldiers then dropped down in a state of insensibility, or coma—others were seized with purely apoplectic symptoms—and all were remarkably relieved by means of blood-letting.

Of the effects of such causes, I saw a still more striking and melancholy example in India, 1797; where an European regiment, little habituated to the climate*, but very much addicted to drunkenness, and quartered

* It has been generally supposed to be a part of a commanding officer's duty, gradually tered in a ſtation where arrack was ſold remarkably cheap—the men, after a night's debauch, were taken out in the morning to be marched a few miles to ſober their ſenſes, as it was called. This was *certainly* neceſſary; for before leaving the parade, many were yet reeling. They were marched a few miles—the ſun roſe—the men lagged—many dropped—ſix were brought in dead—not one fourth of the regiment kept together to the fort—above a hundred were taken to the regimental hoſpital—and ſeveral died in conſequence of being attacked with fever and fluxes. The lives of many were ſaved by the uſe of blood-letting, from being apparently attacked with phrenitis: and this circumſtance calls ſtrongly back to my recollection, the laxity of medical diſcipline which I obſerved at that time, in that part of the world. Under no circumſtances, in my opinion, is the preſence of the medical man more indiſpenſibly neceſſary, than when his regiment is on the march; no pretence whatever ſhould at ſuch times authorize his abſence; in no climate, perhaps, in ſuch caſes may a ſurgeon be more likely to be of real benefit.—The medical allowances granted by the Hon. Eaſt India Company to European regiments are not only remarkably liberal, but in fact unneceſſarily extravagant: the war-allowance for medical attendance for a thouſand men exceeds five thouſand pounds a year! and yet, ſtrange to tell, there was not one medical man, black or white, to be ſeen the whole time the regiment was on its march, at the time the men were falling every minute. If fifty guineas a month are allowed for doolies* on ſuch occaſions, I do not ſee why they ſhould be withheld. If a ſurgeon is allowed beſides, three hundred guineas a month for attending on the ſick, I do not conceive what indulgence he can claim for being abſent. The mind of man becomes in a variety of ways relaxed, and enervated, and peeviſh, and fond of ſenſual gratifications in an Indian climate. *We* know female charms, even in tawny Hindoo colours, in the cool mornings, on the ſhores of Coromandel, or the plains at Columbo, are very faſcinating, very enticing; but ſurely the ſoft murmurs of languiſhment, or the toilſome pleaſures of private duty, ſhould never interfere with that duty which is indeed of an important nature—that duty where the *lives* of our fellow-creatures are at ſtake, and in our hands!

From theſe inſtances, and a number of others which might be mentioned, we find a variety of diſeaſes brought on, ſeemingly by the ſame cauſes, namely, dually to ſeaſon his men to the climate. Youthful European conſtitutions will not bear the fatigues of drilling and marching, till they are accuſtomed to the climate.

* Doolies are a kind of palanquin, or couch, carried by two or more men, in the rears of regiments, while on fatiguing marches, for conveying thoſe men who happen to be taken ill.

namely, exceſſive fatigue, with great heat, occaſioning fever, dyſentery, phrenitis, hepatitis, and apoplexy. Is it probable, that in the two former there was the action of a peculiar contagion? Or are we not rather to ſuppoſe the ſame external cauſes produced all the different diſeaſes?

The nature of all contagions ſeems to be but little underſtood, and what is frequently ſaid on the ſubject appears to me to be involved in much obſcurity; but the bodies of men, placed in certain ſituations and circumſtances, and vegetable matter undergoing certain changes, appear to be capable of generating certain morbific poiſons, occaſioning certain ſpecific diſeaſes, and at times, as I think, without any of the parent poiſon (if the expreſſion will be allowed), being primarily preſent. Contagious fevers and fluxes, appearing frequently in crouded ſituations, and particularly the manner in which the yellow fever broke out in America, ſeem to favour this opinion; while I think it true, that contagions may be evolved from accidental circumſtances, I think it alſo not improbable, that all morbid poiſons may at firſt have ariſen in this manner, and that all may exiſt in effluvium, or a gaſeous ſtate only; and though it appears likely that ſome, as that of ſyphilis, or hydrophobia, &c. are communicated in a palpable or viſible form, others, as that of the ſmall-pox, exiſt only in the gaſeous ſtate, involved and communicated by the medium of different vehicles, namely, the air and purulent matter, and thus giving riſe to the diſeaſe as it appears in the natural and inoculated way. When we ſpeak of the variolous poiſon being communicated in the palpable form, and in the ſtate of effluvium, it conveys to our mind, as if there was ſome difference both in the form and nature of the contagion; and it is ſaid, when the poiſon is communicated in the ſtate of effluvium by the air, or where the diſeaſe appears in the natural way, the ſymptoms of the ſmall-pox are longer in appearing. This is certainly true, but may not the difference ariſe from the manner in which the contagion is applied? When the diſeaſe ariſes in the natural way, I would ſay, the ſymptoms are longer in appearing, becauſe then the poiſon is moſt probably taken in by the lungs or ſkin, and there, or about the throat, meeting and blending with mucous matter on a healthy ſurface, it is for ſome time prevented from being abſorbed, and ſhewing its effects on the conſtitution; while the poiſon, as it is communicated through the medium of pus, and by the puncture of the lancet, immediately applied to the action of the abſorbents, its effects are thus more early produced. Some years ago I remarked the following fact:—a child with a ſore, in conſequence of inflammation on one of the tonſils, was unguardedly taken into a houſe where ſeveral patients were labouring under natural ſmall-pox. The ſymptoms of the ſmall-pox began to appear in the child in nine days thereafter.

Here

Here then we find the difeafe appearing in the ufual time of inoculation. Was this in confequence of the poifon being applied by the atmofphere to an actively abforbing furface—the fore in the throat? Or had the child received the infection before entering the houfe where the difeafe was? Certain it is, however, the healing of the fore on the tonfil was arrefted, became more inflamed and larger during the difeafe, which, upon the whole, was mild. I confider the difference of the violence of fmall-pox to arife chiefly from the quantity of the poifon thrown into the fyftem: perhaps too its being involved in that bland matter, pus, its virulence may in fome meafure be diminifhed, and why inoculation fhould fometimes produce a violent difeafe, may arife from peculiarity of conftitution, heat of the air, and other circumftances. I cannot fuppofe the variolous matter can be at all compared to a ferment, for then inoculation fhould, in every inftance, be attended with as violent a difeafe as that arifing in the common way. The original quantity alone introduced into the fyftem, appears to be evolved and fet into activity by the heat in the fluids of our body, and the poifon may not have the power of generating more, until pus begin to be formed in the puftules;—analogous in fome way, perhaps, to what we obferve in various fubftances, particularly in manganefe, which, when expofed to confiderable heat, gives out great quantities of oxygen gas, and after being entirely robbed of it, will again, on expofure to the air, charge itfelf with the fame matter. In fome parts of Scotland they have a cuftom of taking the variolous matter from the puftule when beginning to form, and they think this in general produces a milder difeafe.

(To be continued in our next Number.)

---

### *To the Editors of the Medical and Phyfical Journal.*

GENTLEMEN,

