# Errata

**Published:** 1799-07

**Authors:** 


No. IV. p. 352, l. 18. for "acid fæces," *acrid* fæces."

———ib. in the note: for "to be a part," read, "to be a *nice* part",

END OF VOL. I.

